# The mystery of unidentified infrared emission bands

**DOI:** 10.1007/s10509-022-04045-6

**Published:** 2022-02-02

**Authors:** Sun Kwok

**Affiliations:** Department of Earth, Ocean, and Atmospheric Sciences, University of British Columbia, Vancouver, Canada

**Keywords:** Astrobiology, Astrochemistry, ISM: lines and bands, ISM: molecules, Planetary nebulae: general, Reflection nebulae, Stars: AGB and post-AGB, Galaxies: starburst

## Abstract

A family of unidentified infrared emission (UIE) bands has been observed throughout the Universe. The current observed spectral properties of the UIE bands are summarized. These properties are discussed in the frameworks of different models of the chemical carriers of these bands. The UIE carriers represent a large reservoir of carbon in the Universe, and play a significant role in the physical and chemical processes in the interstellar medium and galactic environment. A correct identification of the carrier of the UIE bands is needed to use these bands as probes of galactic evolution.

## Discovery of the unidentified infrared emission bands

When astronomical infrared observational capabilities first developed in the 1960s, the expectation was that the infrared spectra of emission nebulae would be dominated by atomic fine-structure lines. The detection of a strong infrared excess in the planetary nebula NGC 7027 was totally unexpected (Gillett et al. 1967). The large infrared continuum emission in NGC 7027 and other planetary nebulae was later identified as thermal emission from circumstellar solid-state grains. Even more surprising was that on top of this strong infrared continuum, there is a strong emission feature at 11.3 μm that cannot be identified with any known atomic lines (Gillett et al. 1973). Further ground-based observations found another strong unidentified emission feature at 3.3 μm in NGC 7027 (Merrill et al. 1975). Russell et al. (1977a) found that the strength of the 11.3 μm feature closely correlates with the 3.3 μm feature, suggesting a common origin for the two features. Since the 3.3 and 11.3 μm features are too broad to be atomic lines and show no substructures to qualify as molecular bands, they were believed to arise from mineral solids such as carbonates (Gillett et al. 1973; Russell et al. 1977a). This seemed a reasonable interpretation at the time because silicate minerals have already been found to be common in the circumstellar envelopes of evolved stars (Woolf and Ney 1969).

As ground-based observations in the infrared are limited by the availability of atmospheric windows, there was a spectral gap between 4–8 μm that remained unexplored. From spectrophotometric observations obtained from the *Kuiper Airborne Observatory (KAO)*, additional features at 6.2, 7.7, and 8.6 μm were discovered (Russell et al. 1977b, 1978). The failure to detect the expected 7.0 μm carbonate feature ruled out the mineral hypothesis. Since the 3.3, 6.2, 7.7, 8.6, and 11.3 μm features have no counterparts in the infrared spectra of atoms, they are collectively known as the unidentified infrared emission (UIE) bands.

The UIE bands are found to be present in planetary nebulae, reflection nebulae, Hii regions, novae, as well as spatially widely distributed in the diffuse interstellar medium in the Milky Way Galaxy and in external galaxies.

In addition to their ubiquitous nature, the most intriguing element of the UIE phenomenon is that it is a manifestation of organic matter in space. Among the first to suggest that there could be an organic component in the interstellar medium was Bertram Donn, who proposed polycyclic hydrocarbons as a possible component of interstellar grains (Donn 1968). After the discovery of the UIE bands, Hoyle and Wickramasinghe (1977) suggested that the spectrum of polysaccharides shows resemblance to the astronomical UIE bands.

The infrared spectra of organic compounds have actually been well studied by chemists since the 1960s. This connection between astronomical spectra and laboratory spectroscopy was made by Knacke (1977), who identified the 3.3 μm UIE band as originating from C-H stretching mode of aromatic compounds. The possible organic origin of the UIE feature were also raised by Puetter et al. (1979). Sagan and Khare (1979) suggested a connection between the astronomical UIE bands and the laboratory-synthesized complex organic polymer tholins (Khare and Sagan 1973). The organic origin of the UIE bands was extensively discussed by Duley and Williams (1981), who assigned the 3.3 and 11.3 μm features to aromatic materials. All these were treated as speculations at the time and received little attention from the astronomical community.

## The UIE phenomenon

**Fig. 1:**
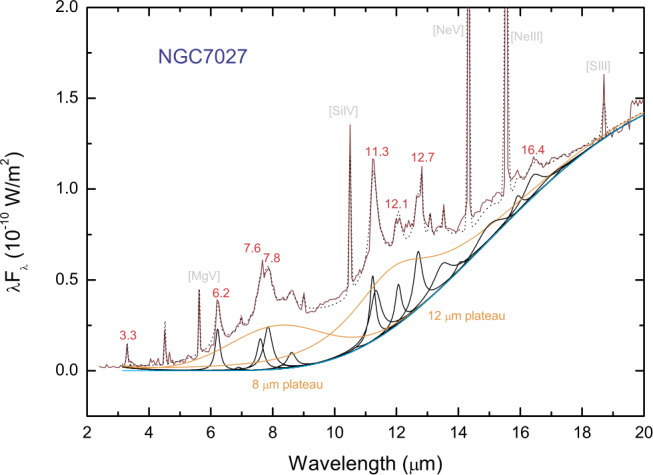
UIE bands in the planetary nebula NGC 7027 as observed by the *Infrared Space Observatory*. The observed spectrum is spectrally decomposed into the UIE bands (in black), plateau features (in orange), and the underlying dust continuum (in blue). The wavelengths of the UIE bands are labeled in red. The narrow features (labeled in grey) are atomic lines. Figure adapted from Kwok and Zhang (2011)

**Fig. 2:**
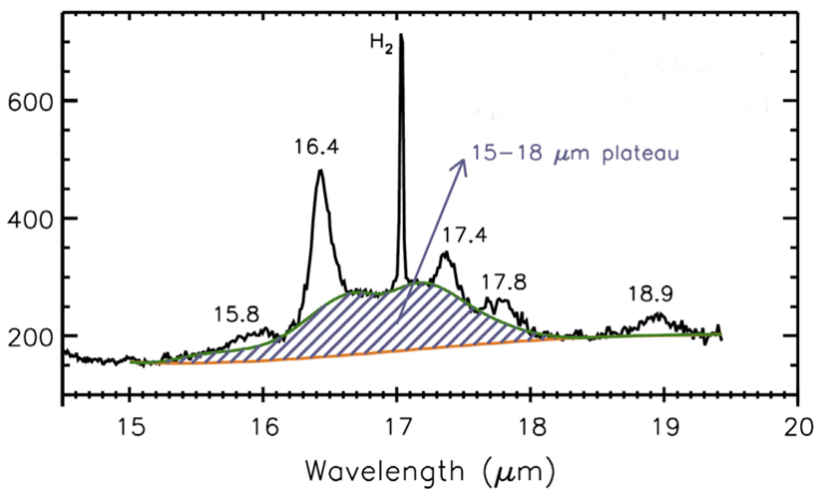
*Spitzer* IRS spectrum of NGC 7023 showing the 15–20 μm broad emission plateau (the shaded region) centered around 17 μm and the minor UIE bands at 15.8, 16.4, 17.4, and 17.8 μm. The 18.9 μm feature is assigned to C_60_. Figure adapted from Peeters et al. (2012)

Although most of the discussions of UIE bands have focused on the strong 3.3, 6.2, 7.7, 8.6, and 11.3 μm bands, the UIE phenomenon is much richer than these features. There are a number of minor emission features at 12.1, 12.4, 12.7, 13.3, 15.8, 16.4, 17.4, 17.8, and 18.9 μm, which have been observed in proto-planetary nebulae (Kwok et al. 1999), reflection nebulae (Sellgren et al. 2007), and galaxies (Sturm et al. 2000) (Figs. 1 and 2). Most significantly, the UIE features are often accompanied by strong, broad emission plateaus features at 6–9, 10–15, and 15–20 μm (Figs. 1 and 2).

### Discovery of aliphatic organics

Motivated by Hoyle’s idea that organic matter is common in interstellar space, Wickramasinghe and Allen (1980) undertook a search for absorption signatures along the sight to the Galactic Center and detected a strong signal at 3.4 μm. This feature is most likely due to C-H stretch of aliphatic compounds, which Duley and Williams (1979) had suggested to be observable through absorption spectroscopy toward infrared sources. This work was followed by a number of studies using both ground-based and space-based telescopes (Pendleton et al. 1994; Sandford et al. 1991; Chiar et al. 2000; Dartois et al. 2004a), leading to the discovery of the aliphatic C-H bending mode at 6.9 μm. The 3.4 μm feature has also been detected in external galaxies (Imanishi 2000; Imanishi et al. 2010) The strength of the 3.4 μm feature suggests that at least 15% of all the carbon atoms are in the form of aliphatic compounds (Dartois 2011)

The same 3.4 μm feature was also found in emission in planetary nebulae showing the 3.3 μm feature (Jourdain de Muizon et al. 1986), which implies that the carriers of UIE bands are not purely aromatic. Although the 3.3 μm band is generally much stronger than the 3.4 μm band, in some sources (e.g., proto-planetary nebulae) the two features can be comparable in strength (Geballe et al. 1992; Goto et al. 2007). Distinct components of the 3.4 μm feature at 3.40, 3.46, 3.52, and 3.56 μm have been attributed to symmetric and anti-symmetric C-H stretching modes (Jourdain de Muizon et al. 1990; Hrivnak et al. 2007).

### Band positions and profiles

**Fig. 3:**
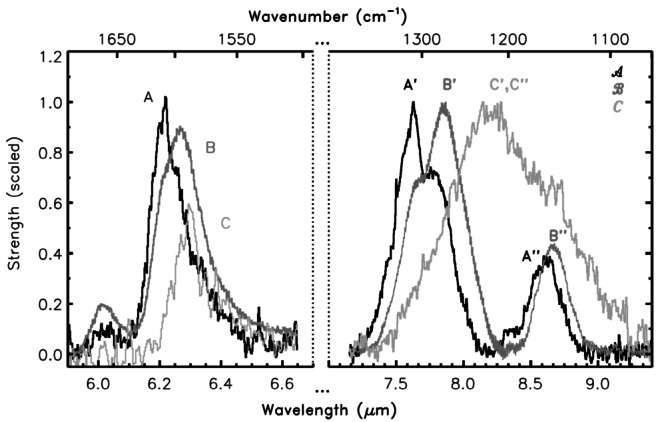
The band position and profiles of the 6.2 μm (left panel) and 7.7/8.6 μm (right panel) features in different classes of objects. Class *A* objects have band peaks at 6.2 (A), 7.6 (A′) and 8.6 μm (A^′′^), Class *B* objects have band peaks at 6.25 (B), 7.8 (B′), and 8.8 μm (B^′′^). Class *C* objects have band peaks at 6.3 (C), broad 8.2 μm (C′ and C^′′^). Figure adapted from Peeters et al. (2002)

The observed astronomical UIE spectra can be classified into different groups based on the band positions and profiles. Peeters et al. (2002) and van Diedenhoven et al. (2004) classified the spectra into classes *A*, *B*, *C*, and *D*, with the 7.7 μm feature showing the largest variation among the classes. The 7.7 μm band shows peak positions at 7.6 and 7.8 μm, and sometimes at 8.0, and 8.2 μm, and these variations are used to designate the different classes (right panel, Fig. 3). The 6.2 μm feature sometimes peaks at wavelength as long as 6.29 μm (left panel, Fig. 3). Generally speaking, Hii regions, reflection nebulae, and galaxies belong to Class *A*, Herbig AeBe stars to class *B*, and post-Asymptotic-Giant-Branch stars to Class *C*. This classification scheme is also applied to UIE sources in the Magellanic Clouds (Sloan et al. 2014).

### Underlying continuum

**Fig. 4:**
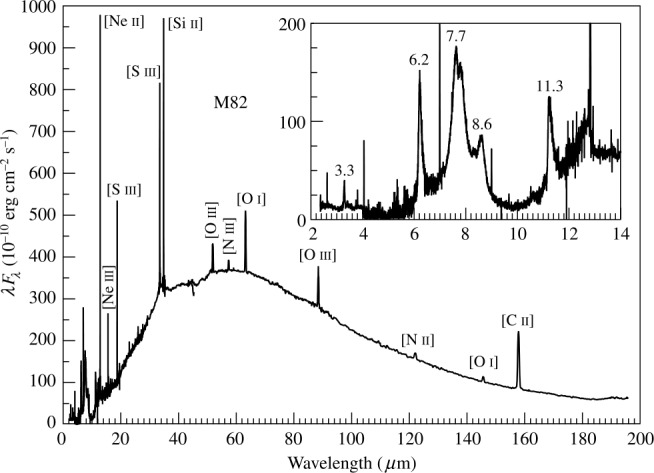
The UIE bands (expanded in insert box) of the starburst galaxy M82 are observed on top of an infrared continuum with a color temperature of ∼50 K. The narrow lines are atomic lines. Figure adapted from Kwok (2007)

The UIE bands are often observed in emission on top of a continuum (Figs. 1 and 4). This continuum is attributed to thermal emission from micron-size solid particles (“dust” in astronomical nomenclature). Since this continuum is often featureless, it is assumed to originate from amorphous carbon grains. In the diffuse interstellar medium, the strengths of the UIE bands are correlated with the strength of the dust continuum (Kahanpää et al. 2003). Such correlations imply that the UIE bands and the dust continuum must share the same heating source.

### Association with fullerenes

Since the initial discovery of fullerene (C_60_) in the planetary nebula Tc-1 (Cami et al. 2010), C_60_ emissions have been detected in reflection nebulae (Sellgren et al. 2010), planetary nebulae (García-Hernández et al. 2010), and proto-planetary nebulae (Zhang and Kwok 2011) where UIE bands are simultaneously present. Although Tc-1 has no strong UIE features, it does show the broad 8 and 12 μm plateaus features. The association between C_60_ and the 8 and 12 μm plateau features (Zhang and Kwok 2013; Otsuka et al. 2013) suggests that amorphous carbonaceous solids could be precursors of fullerenes (García-Hernández et al. 2012; Bernard-Salas et al. 2012).

## Chemical nature of the carriers

Although the carrier of the UIE bands are generally recognized as due to a carbonaceous compound since the work of Duley and Williams (1981), the exact chemical composition and structure of the carrier is not yet settled. Some of the models are discussed below.

### Polycyclic aromatic hydrocarbon molecules

The idea that UIE bands originate from polycyclic aromatic hydrocarbon (PAH) molecules was proposed by Léger and Puget (1984) and Allamandola et al. (1985). Allamandola et al. (1985) calculated the vibrational spectrum of gas-phase chrysene and suggest possible matches to the UIE bands. The PAH hypothesis was developed based on (i) some general similarities between the infrared spectra of UIE bands with PAH molecules; and (ii) the 12 μm excess emission observed in cirrus clouds in the diffuse interstellar medium can be explained by single-photon excitation of small molecules (Sellgren 1984, 2001). The physics of PAH molecules as a component of the interstellar medium was discussed by Omont (1986).

The thesis of the PAH hypothesis is summarized by Tielens (2008) as “These features are (almost) universally attributed to the IR fluorescence of far-ultraviolet (FUV)-pumped polycyclic aromatic hydrocarbon (PAH) molecules, containing 50 C atoms”. The small size of the PAH molecules allow them to be stochastically excited to temperature of 1,000 K upon absorption of a single UV photon, and subsequent cascade produces the vibrational emission bands in the infrared. In the past 30 years, the PAH hypothesis has become extremely popular and is commonly accepted in the astronomical community as the explanation of the UIE phenomenon. The 3.3 μm UIE band is assigned to C-H stretching modes, the 6.2 μm and 7.7 μm features to C-C stretching modes, the 8.6 μm feature to C-H in-plane bending modes, and the 11.3 μm to C-H out-of-plane bending modes of PAH molecules (Allamandola et al. 1989). Details of the PAH hypothesis are reviewed by Peeters (2011) and Peeters et al. (2021). The application of PAH hypothesis to the study of galaxies is reviewed by Li (2020). The spectral properties of PAH molecules have been extensively studied (Hudgins and Allamandola 2004; Salama 2008) and collected into a comprehensive database (Bauschlicher et al. 2010; Boersma et al. 2014; Mattioda et al. 2020).

The first serious objections to the PAH hypothesis was given by Donn et al. (1989). Below is a list of problems with the PAH hypothesis, expanded and updated from the list of Donn et al. (1989).

**Fig. 5:**
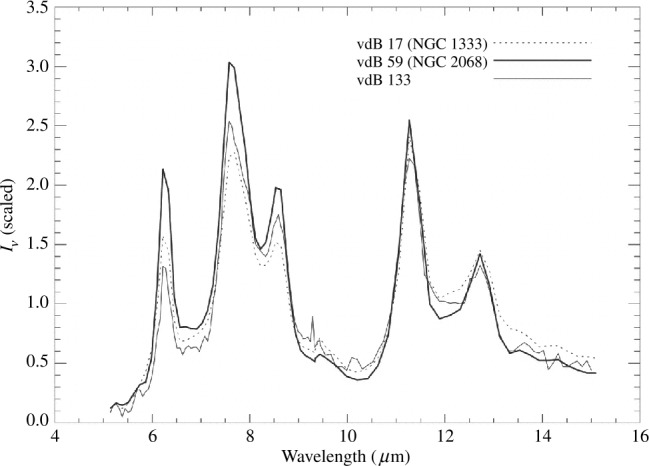
Normalized *Infrared Space Observatory* spectra of three reflection nebulae showing that the UIE bands have similar peak wavelengths, spectral shapes, and continuum levels independent of the temperatures of the illuminating stars (vdB 17, 11,000 K; vdB 59, 19,000 K; vdB 133 6,800 K). Figure adapted from Uchida et al. (2000)
- Presence of aliphatic features in UIE sources. In the PAH hypothesis, the 3.4 μm feature is interpreted as superhydrogenation of PAH molecules (Schutte et al. 1993). It has also been suggested as arising from hot bands from anharmonic aromatic C-H stretch, which shifts the 3.3 μm feature to a longer wavelength (Barker et al. 1987). However, theoretical calculations including anharmonicity show a simultaneous increase of the width of the 3.3 μm feature, which was not observed (van Diedenhoven et al. 2004). Furthermore, the expected strong overtone bands at the 1.6–1.8 μm region is not detected, making the anharmonicity explanation unlikely (Goto et al. 2003).

- PAH molecules have well-defined sharp absorption bands but the UIE bands are broad (Figs. 1 and 3). Superpositions of vibrational bands from a large variety of PAH molecules and artificial broadening profiles have to be introduced to fit the astronomical spectra (Li and Draine 2001; Draine and Li 2007; Cami 2011).
- Neutral PAH molecules are primarily excited by UV and have little absorption in the visible (Ciar 1964; Léger et al. 1989), but UIE features are seen in proto-planetary nebulae and reflection nebulae with no background UV radiation.
- The expected strong absorption features of PAH molecules in the UV are not seen in interstellar extinction curves. The observed PAH to hydrogen abundance ratio upper limits range from $10^{-10}$ to $10^{-8}$ (Clayton et al. 2003; Salama et al. 2011; Gredel et al. 2011), which are much lower than the PAH/H ratio of $3\times 10^{-7}$ predicted from the strength of the infrared features (Tielens 2008).
- The infrared spectra of small PAH molecules are well studied by chemists (Schlemmer et al. 1994; Cook et al. 1996; Cook and Saykally 1998) and they found “No PAH emission spectrum has been able to reproduce the UIE spectrum w.r.t. either band positions or relative intensities” (Wagner et al. 2000).
- The PAH model predicts that the UIE band ratios are strongly dependent on the UV background as individual UIE bands are assumed to arise from different neutral or ionized PAHs (Hudgins and Allamandola 2004; Draine et al. 2021). But the shapes and peak wavelengths of UIE bands are found to be independent of temperature of exciting star, ranging from thousands to tens-of-thousands of degrees (Uchida et al. 2000) (Fig. 5). An analysis of 820 UIE spectra in the diffuse interstellar medium shows no variation of the UIE band ratios in regions of widely different UV backgrounds (Chan et al. 2001).

An independent way to establish the presence of PAH molecules in space is to search for them through their rotational transitions. However, due to the absence of permanent dipole moment of these molecules, this is not as feasible as for other classes of molecules. The simplest ring molecule benzene is detected via its infrared transitions in the proto-planetary nebula AFGL 618 (Cernicharo et al. 2001). Two double-ring molecules with nitrile functional groups (1- and 2-cyanonaphthalene, $c$-C_6_H_5_CN) have been detected in the molecular cloud TMC-1 (McGuire et al. 2021). Two pure hydrocarbon cyclic molecules: cyclopentadiene ($c$-C_5_H_6_, a 5-ring molecule) and indene ($c$-C_9_H_8_, a bicyclic molecule with both a 5- and 6-membered ring) have been detected in TMC-1 (Cernicharo et al. 2021; Burkhardt et al. 2021).

In part to address the above list of problems, the PAH hypothesis has been revised to incorporate different ionization states and large sizes to increase the absorption cross sections in the visible. The molecular size range has been extended to >1,000 atoms (Peeters et al. 2021). The PAH molecules are also modified to include dehydrogenation, superhydrogenation and minor aliphatic side groups (Li and Draine 2012). Heteroatoms (defined as those atoms other than C or H) such as N are also introduced to explain the 6.2 μm band (Hudgins and Allamandola 1999).

In order to fit the astronomical observations, the PAH model appeals to a mixture of PAH of different sizes, structures (compact, linear, branched) and ionization states, as well as artificial broad intrinsic line profiles (Allamandola et al. 1999; Cami 2011). The number of free parameters is so large that the routines used by the PAH model to fit the astronomical UIE spectra have been shown to be able to fit any artificial spectra (Zhang and Kwok 2015).

### Other hydrocarbons

**Fig. 6:**
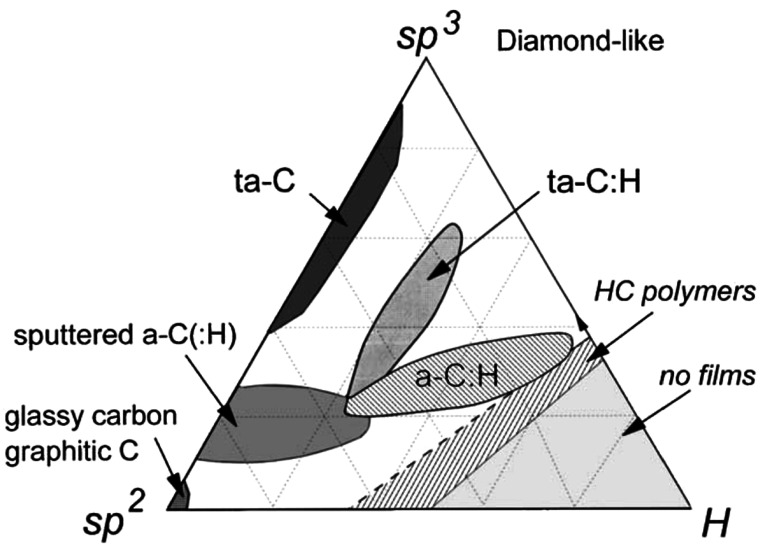
Phase diagram of amorphous carbonaceous (pure C and H) compounds. The lower right corner of the triangle represents pure H, lower left corner pure graphite-like ($sp^{2}$) materials, and the upper corner pure diamond-like ($sp^{3}$). Areas inside the triangle represent various H/C ratios and $sp^{2}/sp^{3}$ mixed hybridization states. Figure adapted from Robertson (2002)

In addition to pure carbon materials such as graphite (hybridization state $sp^{2}$) and diamond (hybridization state $sp^{3}$), different amorphous C-H alloys can be created when H is introduced (Cataldo 2004; Jones et al. 2013). These amorphous solids consist of varying degrees of aromatic to aliphatic ratios, number of aromatic rings, different lengths of aliphatic chains, and arranged in different geometric structures. A schematic of possible structures of amorphous carbonaceous solids is shown in Fig. 6. The lower left corner of the triangle represents graphite (C rings on a plane with no H), the top corner represents diamonds (C arranged in tetrahedral forms), PAHs (aromatic rings arranged on a plane) are on the bottom edge, and various forms of amorphous hydrogenated carbon can exist in the interior of the triangle. Theoretical calculation on the infrared spectra of such amorphous solids suggest that they can be good candidates as carrier of UIE bands (Jones 2012a,b). Since these amorphous carbonaceous solids have absorption bands in the visible, they can be easily excited by visible light from stars.

The possibility that the UIE bands could be due to some form of hydrogenated amorphous carbon (HAC) was made by Buss et al. (1990), and HAC was proposed to be a major constituent of interstellar dust by Jones et al. (1990). Further work on HAC was described in Duley (1993) and Duley (2000). The infrared properties of HAC were studied in the laboratory by Scott et al. (1997), Duley et al. (2005), and Gadallah et al. (2012).

By subjecting methane gas in a vacuum to microwave radiation, Sakata et al. (1983) were able to collect carbonaceous composite particles on a substrate after rapid cooling, which they name quenched carbonaceous composite (QCC). The infrared spectra of QCC show strong resemblance to the astronomical UIE bands (Sakata et al. 1984, 1987).

By accreting C, C_2_, and other C_*n*_ molecules, carbon nanoparticles with sizes less than 100 nm can be created in the laboratory. The infrared spectra of these particles show similarities to the astronomical UIE spectra (Hu et al. 2006; Hu and Duley 2008).

### Amorphous hydrocarbons with heteroatoms

**Fig. 7:**
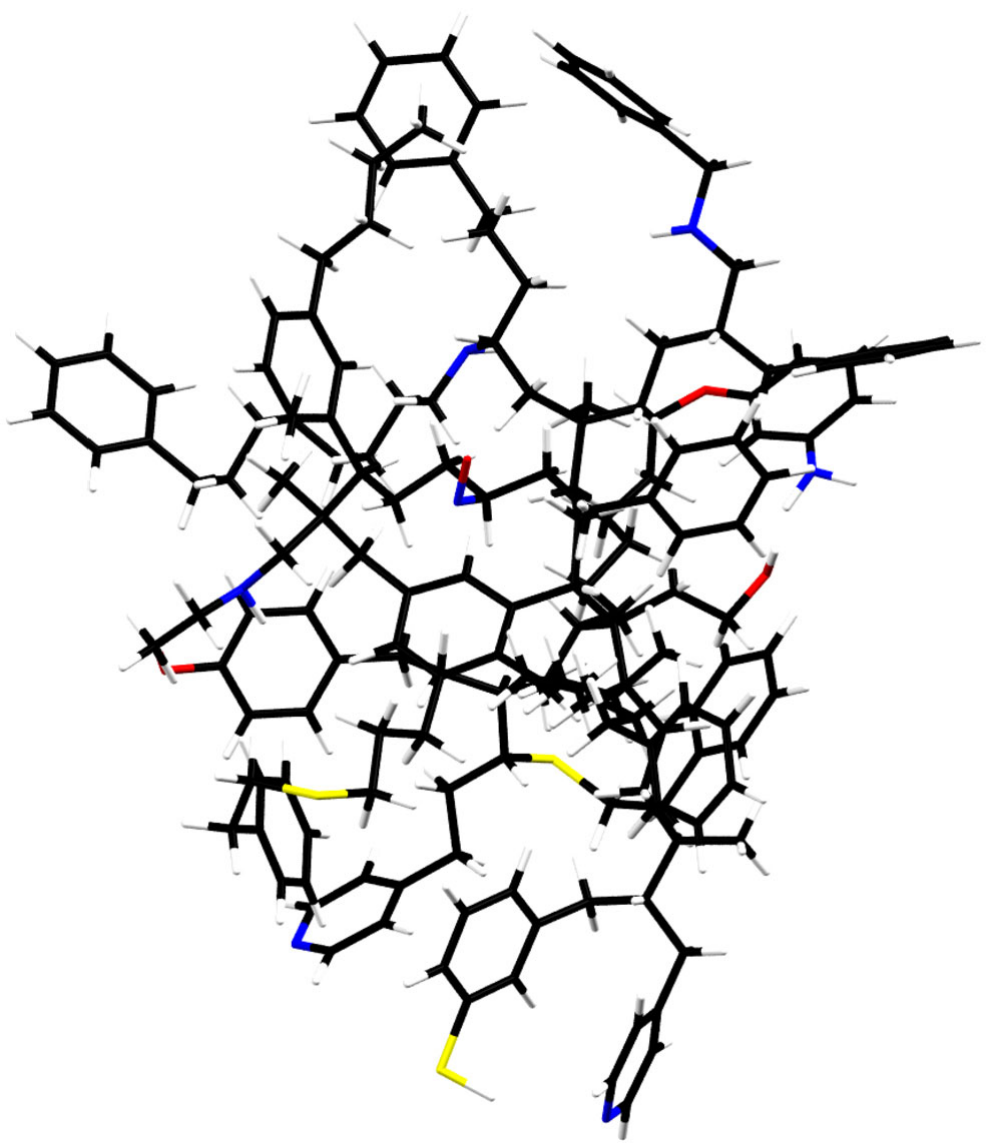
The MAON structure is characterized by a highly disorganized arrangement of small units of aromatic rings linked by aliphatic chains. This structure contains 169 C atoms (in black) and 225 H atoms (in white). Impurities such as O (in red), N (in blue), and S (in yellow) are also present. A typical MAON particle may consist of multiple structures similar to this one

Mixed aromatic/aliphatic structures are natural products of combustion. The first nucleation products of soot particles formed in flames have structures consisting of islands of aromatic rings linked by chains (Colangeli et al. 1997; Chung and Violi 2011). In natural interstellar or circumstellar environments, other elements such as O, N, and S are abundantly present and can be expected to be incorporated into any carbonaceous compounds condensed from gas phase. Based on the similarities between the astronomical UIE spectra and the infrared spectra of coal, Papoular et al. (1989) suggested that coal as the carriers of the UIE bands. This idea was later extended to kerogen-like materials (Papoular 2001). Petroleum fractions as a carrier of UIE bands are discussed by Cataldo et al. (2002). Coal, kerogen, and petroleom are natural products that contain heteroatoms in their structures. The model of mixed aromatic/aliphatic organic nanoparticles (MAON) considers a carbonaceous compound containing aromatic rings of different sizes and aliphatic chains of different lengths and orientations arranged in a 3-D amorphous structure, mixed with heavy elements (Kwok and Zhang 2013). Figure 7 shows a schematic structure of MAON, illustrating the complexity of the chemical structure, as well as the kinds of functional groups (including heteroatoms) that may be contained in such structures.

By introducing nitrogen gas into the QCC experiment, a solid condensate quenched nitrogen-included carbonaceous composite (QNCC) is collected. The infrared spectra of QNCC show resemblance of the UIE spectra of novae (Endo et al. 2021).

## Other synthesized substances

Since the early work on tholins (Khare and Sagan 1973), various carbonaceous compounds have been synthesized in the laboratory by subjecting simple hydrocarbons to different forms of energy injections. The techniques used include laser ablation of graphite (Scott and Duley 1996; Mennella et al. 1999; Jäger et al. 2008), laser pyrolysis of gases (Herlin et al. 1998), arc discharge (Mennella et al. 2003), microwave irradiation (Sakata et al. 1984; Wada et al. 2009; Godard et al. 2011), UV photolysis (Dartois et al. 2004b), and flame synthesis (Carpentier et al. 2012). Chemical analysis of these laboratory synthesized carbonaceous nanoparticles show that they consist of networks of chains and rings (Hu et al. 2006) and their spectra show varying degrees of resemblance to the astronomical UIE spectra.

## Atomic origin

The first proposal for an atomic origin of the UIE bands was made by Holmlid (2000) who suggests that the astronomical bands are the result of electronic deexcitation of Rydberg matter states with principal quantum numbers $n=40-200$. Rydberg matter is proposed to be a new phase of matter equivalent to that of liquid and solid which could exist in space in large quantities. In support of this hypothesis, Holmlid (2000) shows that the Rydberg matter model can fit the observed UIE spectra with fewer parameters than the PAH model. This idea was extended by Zagury (2021a) who assigns the 3.3, 6.2, and 11.3 μm UIE bands to hydrogen recombination line series $n=6$, 8, and 11, respectively, and the 15-20 μm plateau emission band to the continua of hydrogen transitions to $n=13$ and $n=14$. However, the hydrogen hypothesis is contradicted by the fact that the UIE phenomenon is observed only in carbon-rich sources (Cohen et al. 1986, 1989).

## Corresponding vibrational modes of the UIE features

Since the UIE phenomenon consists of a family of emission features, a successful theory must be able to consistently explain the positions, profiles, relative strengths of all the entire family. The ideal candidate for the carrier of UIE bands should consistently produce the entire family of UIE bands without appealing to special conditions. PAH molecules show spectral features near 3.3 and 11.3 μm but do not have clear counterparts for the 6.2, 7.7, and 8.6 μm UIE bands. In the words of Cook and Saykally (1998): “In order to reproduce the narrow 6.2 and 11.2 μm UIR bands, the carriers must consistently exhibit bands at these positions with a consistency similar to that which is observed with the 3.3 μm emission. In addition, the carriers of the UIRs must, in general, exhibit an absence of strong bands in the gap between the 6.2 and 7.7 μm UIR features. The PAHs used in these model spectra simply do not meet these criteria; hence they do not reproduce the details of the UIR spectra.”

One of the advantages of the HAC/MAON-type of models is that they naturally produce broad infrared features without appealing to artificial fittings (Dischler et al. 1983; Guillois et al. 1996). The exact positions of the bands depend on the composition of the material (C to H ratio, fraction of impurities), aromatic to aliphatic ratios, and geometric factors, but their resemblance to the astronomical UIE bands is unmistakable (Fig. 9, Kwok 2016). However, there is no specific vibrational mode assignments to these observed bands and the molecular dynamical origin of the strong bands observed in laboratory specimens (e.g., those observed in Dischler et al. 1983; Herlin et al. 1998) are not identified. In order to advance the MAON hypothesis further, we need to perform quantum chemistry calculations of large varieties of MAONs to identify the major vibrational modes as well as to see whether some of these modes will converge to resemble the observed astronomical UIE bands (Sadjadi et al. 2016).

### The 3.3 μm band

**Fig. 8:**
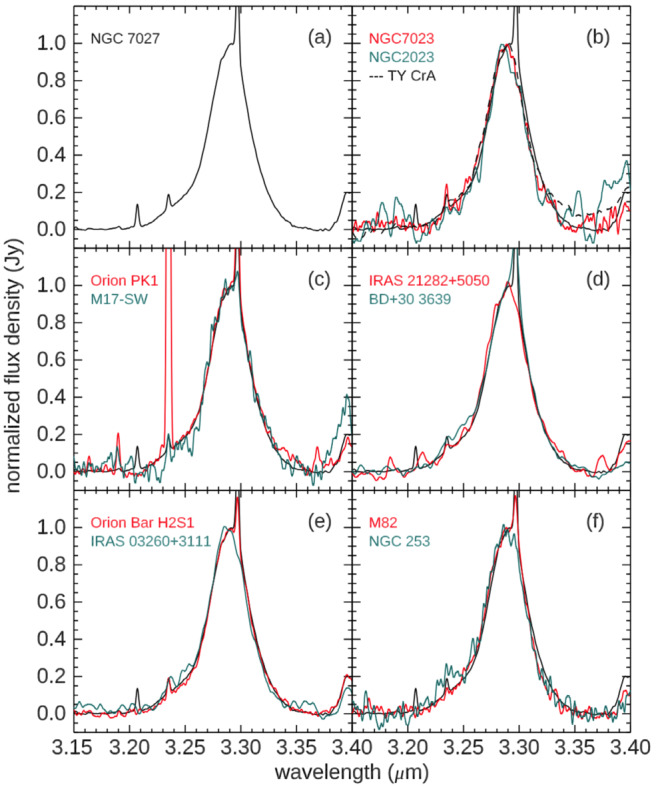
Profiles of the 3.3 μm feature observed in NGC 7027 (plotted in black in all panels) and other astronomical sources (plotted in red and green in panels b-f, as well as in black dashed line in panel b). The sharp lines at 3.207, 3.234, and 3.297 μm are the [Ca IV], H_2_ 1−0 O(5), and H Pfund-$\delta $ lines, respectively. Figure adapted from Tokunaga and Bernstein (2021)

The 3.3 μm UIE feature was first identified as aromatic C-H stretch by Knacke (1977). This is a prominent band and shows a consistent emission profile in many astronomical sources (Fig. 8). The measured central wavelength of the feature is 3.2887±0.0009 μm (Tokunaga and Bernstein 2021). The invariance in feature profile, independent of source excitation conditions and evolutionary history, puts severe constraints on the chemical composition of the carrier and excitation mechanism. The central wavelengths of the C-H stretching mode of PAH molecules lie shortward of the observed wavelength of the 3.3 μm band (Sakata et al. 1990; Kwok and Zhang 2013). Joblin et al. (1995) consider the effects of anharmonic couplings and suggest that the astronomical 3.3 μm feature can be consistent with PAH molecules at high temperatures. But the observed peak wavelengths of the 3.3 μm feature lie within a very narrow range, even when observed in circumstellar or interstellar environments under very different temperature conditions. This makes it unlikely that red shift by high temperatures be the general cause of this wavelength discrepancy.

Ground-based high-spectral resolution observations of the 3.3 μm of HD44179 (the Red Rectangle) suggest that the 3.3 μm feature can be decomposed into two components, one at 3.28 μm and another at 3.30 μm (Song et al. 2003). The existence of these two components has been attributed to “bay” and “non-bay” hydrogen sites of the PAH units, based on the detection of two bands within the range of 3.33-3.21 μm in the experimental gas phase infrared spectra of some small PAH molecules with bay-type hydrogens in their molecular structures. The aromatic C-H stretching mode of PAH has slightly different frequencies depending on how many of the edge H atoms are in “bay” or “non-bay” configurations (Bauschlicher et al. 2009) and it is possible to separate these components by imaging spectroscopic observations (Candian et al. 2012). However, the peak wavelengths of the “bay” and “non-bay” modes are at 3.17–3.27 and 3.26–3.27 μm, respectively, both shortward of the astronomical wavelengths of the 3.28 and 3.30 μm components derived by Candian et al. (2012) by fitting the asymmetric profile of the 3.3 μm feature.

The aromatic C-H stretch at 3.3 μm and aliphatic C-H stretch at 3.4 μm is commonly seen in many carbonaceous compounds (Cataldo et al. 2020). There are also olefinic C-H stretching modes in this wavelength region. For example, Chiar et al. (2013) interpret the 3.28 μm component as the stretching mode of olefinic C-H bonds in amorphous hydrocarbons. The possibility that the 3.30 μm subfeature is due to olefinic C-H stretch is discussed by Sadjadi et al. (2017).

### The 6.2, 7.7, and 8.6 μm bands

Based on a comparison between the astronomical UIE spectra with the laboratory spectrum of coronene, Léger and Puget (1984) assigns the 6.2 μm band to C–C stretch and the 8.6 μm to C–H in-plane bending mode. However, the origin of the 7.7 μm feature is not clear (Allamandola et al. 1985). From the laboratory spectrum of coronene, the 7.4 and 6.4 μm features are suggested to be the combination of aromatic C-C stretch and C-H in plane bending modes (Langhoff 1996). In later discussions under the PAH hypothesis, infrared bands between 6.1 and 6.5 μm are suggested to be due to pure aromatic C-C stretching modes, bands from 6.5 to 8.5 μm are due to coupled C–C stretching and C–H in-plane bending modes, and bands between 8.3 to 8.9 μm are due to C–H in-plane bending modes (Peeters 2011). Since the peak wavelengths of PAH features shift with size of the molecule, the wavelength difference between 6.2 and 7.7 μm features have been used to infer the size of the PAH molecules (Hudgins and Allamandola 1999; Bauschlicher et al. 2008, 2009).

**Fig. 9:**
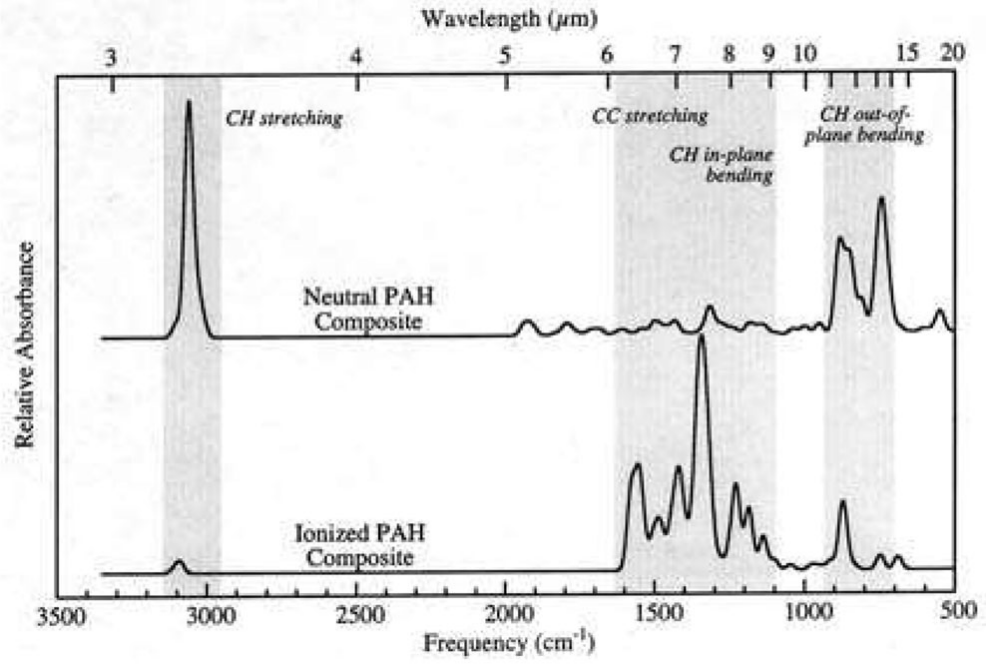
A comparison of the absorption band strengths between neutral (top curve) and ionized (bottom curve) PAH molecules. The UIE bands in the 6−9 μm region are attributed to ionized PAH molecules in the PAH hypothesis. Figure adapted from Hudgins and Allamandola (2004)

However, all C–C stretching modes are very weak in comparison to the C–H modes in neutral PAH molecules. In order to explain the prominence of the features, PAH ions are suggested to be responsible for the UIE features in the 6−9 μm region (Hudgins and Allamandola 2004) (Fig. 9). The need by the PAH hypothesis to simultaneously rely on neutral PAHs to explain the 3.3 and 11.3 μm features and PAH ions to explain the 6.2, 7.7, and 8.6 μm features poses difficulties in explaining the near universal presence of all these features in astronomical sources of very different UV backgrounds (Fig. 5).

As the molecular structure gets more complex (e.g. in MAONs), many of the vibrational modes in the 6–10 μm region become coupled and cannot be identified as a single vibrational mode (Sadjadi et al. 2016).

### The 11.3 μm band

Soon after the discovery of the UIE bands, the 11.3 μm feature was identified as C–H out-of-plane bending mode of aromatic compounds (Duley and Williams 1981). The observed peak position of the 11.3 μm feature in astronomical sources is well defined and does not vary much in wavelength. The 11.3 μm feature is also detected in absorption (Bregman et al. 2000), and its wavelengths and profiles closely resemble those seen in emission. The 11.3 μm feature has a distinctive asymmetric profile, having a steep decline in the short wavelength side and a gradual extended wing in the long wavelength side (van Diedenhoven et al. 2004). Such asymmetric profiles are difficult to explain by gas-phase molecular emissions. In order to explain the observed profile of the 11.3 μm feature in the PAH hypothesis, PAH molecules of different mass are needed: high-mass PAHs will produce the short-wavelength side and low-mass PAHs the long-wavelength side (Candian and Sarre 2015).

Furthermore, the peak positions of the C-H out-of-plane bending modes of PAH molecules can be quite different due to mode coupling, molecular structure, and charge states of the molecules (Hony et al. 2001). Because of the large wavelength variations of the out-of-plane bending modes of PAH molecules, it is difficult to assign or match the observed astronomical feature to specific PAH molecules. Ring deformation vibrational mode of small carbonaceous molecules such as ethylene oxide ($c$-C_2_H_4_O) has also been proposed to explain the narrow 11.3 μm feature (Bernstein and Lynch 2009).

Sadjadi et al. (2015) show that a mixture of pure PAH molecules, even including units of different sizes, geometry and charged states, is unable to fit the astronomical spectra. In order to fit the astronomical profile of the 11.3 μm feature, oxygen-containing molecules are needed.

### Plateau features

The 8 and 12 μm plateau features were first detected in proto-planetary nebulae by *IRAS* LRS and *KAO* observations (Kwok et al. 1989; Buss et al. 1990). Li and Draine (2012) assign these two plateau features as “wings of C–C and C–H bands” of PAH molecular vibrations. If this is the case, then similar “wings” should be seen around the 3.3 μm feature but it is not generally observed. Alternatively, these plateau features have been identified as superpositions of in-plane and out-of-plane bending modes emitted by a mixture of aliphatic side groups attached to aromatic rings (Kwok et al. 2001).

The 15–20 μm plateau feature has been detected in young stellar objects, compact HII regions, and planetary nebulae, and is especially strong in some proto-planetary nebulae (Zhang et al. 2010). The wavelength region of this broad feature suggests that it could arise from C–C skeleton vibrational modes (Van Kerckhoven et al. 2000).

## Synthesis pathways

The circumstellar envelopes of evolved stars (from AGB stars through proto-planetary nebulae to planetary nebulae) are ideal laboratories to test models of molecular synthesis. The sequential formation of molecules from C_2_, C_3_, CN, to HCN, HC_3_N, HC_5_N, C_2_H_2_, to C_6_H_6_ suggests a bottom-up scenario of molecular synthesis. PAH molecules can be the products of molecular synthesis (Frenklach and Feigelson 1989). The linking of islands of aromatic rings by aliphatic chains can lead to formation of MAONs. Fullerenes could be results of bottom-up processes (e.g., from SiC grains, Bernal et al. 2019) or top-down process through the break up of MAONs (García-Hernández et al. 2012; Bernard-Salas et al. 2012; Micelotta et al. 2012).

The chemical time scales of molecular synthesis in the circumstellar environment is constrained by the dynamical time scales of expanding circumstellar envelopes. Gas-phase molecules and inorganic mineral solids are known to be forming in the circumstellar envelopes of AGB stars and planetary nebulae under low density and over very short time scales (Kwok 2004). In proto-planetary nebulae, UIE bands are observed to emerge over time scales of ∼10^3^ years (Kwok et al. 1999). Infrared spectroscopic observations of novae following their outburst show that UIE bands emerge over time scales of weeks (Helton et al. 2011). In nova V705 Cas, the UIE bands, including a strong 3.4 μm feature, were seen within one year after outburst (Evans et al. 2005). These observations suggest that the abiotic synthesis of complex organics is extremely efficient in the circumstellar environment.

After their synthesis in circumstellar envelopes and their ejection into the interstellar medium, there is also a question of whether the carriers of UIE bands (whether they are PAH molecules or MAONs) can survive their journeys through the interstellar medium. The aliphatic side groups in MAONs could be broken off by interstellar background radiation. If multiple units of MAONs aggregate together they may resemble the macroscopic carbonaceous solids similar to the insoluble organic matter (IOM) observed in meteorites (Cody et al. 2011). Such solids are sturdy and are less likely to be destroyed by interstellar processes. Isotope anomalies in IOM suggest that it is probably of interstellar origin (Busemann et al. 2006).

## Size and energetics

One of the key questions about the carriers of the UIE bands is whether they are free-flying gas-phase molecules, nanoparticles, or solids. Whereas the spectral properties of molecules (in particular PAH molecules) are relatively simple and well studied, the properties of nanoparticles consisting of $10^{2}-10^{3}$ heavy atoms cannot be easily extrapolated from bulk materials because of quantum surface effects.

The astronomical UIE bands in planetary nebulae, Hii regions, and galaxies are observed to lie on top of an infrared continuum of color temperature ∼10^2^ K (Fig. 4). In the diffuse interstellar medium, the color temperature of the infrared continuum is even lower. Since the 3.3 μm band lies shortward (on the Wien’s side) of the peak of the infrared continuum, it is unlikely that the 3.3 μm band is excited thermally. Instead, stochastic heating by a single photon, exciting the carrier temporarily to a high temperature, is suggested to be the excitation mechanism (Sellgren 1984; Omont 1986).

An alternate model of chemical heating was proposed by Duley and Williams (2011), who suggest that this process can heat carbonaceous solid particles of sizes from 5-100 nm to emit the 3.3 μm band. The release of H_2_ molecules from chemical heating is also consistent with the correlation between observed 2.18 μm H_2_ emission with the 3.3 μm band.

If stochastic heating is indeed the excitation mechanism of UIE bands, then the sizes of the carrier must be limited to molecules or nanoparticles. If chemical heating can work, then the size limit can be extended upwards. In either case, we still have to work out the relationship between the UIE carriers and the micron-size grains that contribute to the underlying continuum (Sect. 2.3).

## The need for a correct identification of the origin of the UIE bands

Since the UIE bands are commonly used as a tracer of galactic evolution, it is pertinent that we correctly identify the chemical nature of the carrier of the bands. The UIE bands are detected in galaxies with redshifts >4 (Riechers et al. 2014; Armus et al. 2020) and are particularly prominent in ultraluminuous infrared galaxies. The power emitted in the UIE bands can be as high as 20% of the total energy output of these galaxies (Smith et al. 2007). Given the nearly invariance of the band positions, the UIE bands have been used as redshift indicators (Elbaz et al. 2005). In nearby galaxies, the distribution of the UIE bands can be mapped. The *AKARI* satellite has mapped the distribution of the 3.3 and 3.4 μm bands, showing emission from aromatic and aliphatic species are present in the halo of M82, as far as 2 kpc from the galactic center (Yamagishi et al. 2012).

Under the assumption that UIE bands are PAH molecules excited by UV photons, the UIE bands have been used as tracers of star formation in galaxies (see e.g., Smith et al. 2007; Galliano et al. 2008; Wu et al. 2010; Li 2020) as well as the star formation activities as a fraction of the total infrared luminosities of galaxies (Peeters et al. 2004; Spoon et al. 2004). Under similar assumptions, the UIE bands have also been used as tracers of elemental and chemical evolution of galaxies (Genzel et al. 1998).

Based on the assumption that the 11.3 μm feature originates from neutral and large PAHs, and the 6.2, 7.7, and 8.6 μm features originate from PAH ions, the strength ratios between the 11.3 μm feature and 6 to 9 μm UIE features have been used to estimate the properties of the background radiation fields (Galliano et al. 2008; Berné et al. 2009). These results are used to determine the star formation rates of galaxies (Calzetti 2011). The physical validity of these studies, however, depends on a correct interpretation of the origin of the UIE bands.

## Conclusions

The family of astronomical UIE bands is a rich spectral phenomenon which carrier is yet to be unambiguously identified. It is likely to be a carbonaceous substance whose structure is more complicated than it is often assumed. Although the PAH hypothesis has been popular in the astronomical community, its definition has been evolving from the original chemical definition of planar, pure carbon and hydrogen ring molecules, to a collection of molecules including “PAHs with side groups, heterosubstituted PAHs, fully or partially (de)hydrogenated PAHs, and PAH clusters.” (Peeters et al. 2021). This revision of definition is moving the PAH hypothesis closer to other models of the UIE bands.

The debate between the PAH and HAC/MAON models is more than a question of semantics as there are fundamental differences between these two models: Do the UIE carriers contain pure rings or mixed rings and chains? Are their geometry 2-D or 3-D? Are they pure C-H compounds or contain impurities? Are their structure regular or amorphous? Are the carriers gas-phase free-flying molecules or solids? If answers to these questions are toward the latter, we have to call these carriers by their proper chemical terminology.

Since the UIE phenomenon is seen throughout the Universe, even during its early epochs, a correct identification of the carrier is of great importance. Due to the strengths of the UIE bands, the carrier represents a major reservoir of carbon. If the carrier of UIE bands are PAH molecules, they would contain ∼10% of cosmic carbon (Peeters et al. 2021). Many current models of the interstellar medium are based the premise that PAH molecules are the dominant factors in photoelectric heating of interstellar gas and in the ionization balance inside molecular clouds. Whether the carriers of the UIE bands are a collection of free-flying gas-phase PAH molecules or complex organic solid particles has significantly different implications on our understanding of cosmic chemical synthesis, energy exchange between stars and the interstellar medium, and galactic chemical enrichment. Further computational and experimental studies of the vibrational properties of amorphous carbonaceous compounds are needed to solve the UIE mystery.
